# Correlational analysis between neutrophil granulocyte levels and osteonecrosis of the femoral head

**DOI:** 10.1186/s12891-019-2778-7

**Published:** 2019-08-31

**Authors:** Jiang Jiang, Xuqiang Liu, Baojian Lai, Dengjiong Hu, Lizhen Lai, Jiaxiang Xu, Songqing Chen, Xiaofeng Li

**Affiliations:** Department of Orthopedics, the First Affiliated Hospital of Nanchang University, Artificial Joints Engineering and Technology Research Center of Jiangxi Province, No. 17 Yongwaizheng Street, Nanchang City, Jiangxi Province China

**Keywords:** Femoral head necrosis, Neutrophil, Osteonecrosis, Influencing factor, Bone repair

## Abstract

**Background:**

The correlation between peripheral blood neutrophil level and osteonecrosis of the femoral head (ONFH) has not been extensively studied. Thus, we aimed to investigate the correlation between neutrophil level in the peripheral blood (neutrophil granulocyte) and ONFH.

**Methods:**

A total of 984 cases of ONFH and femoral neck fractures (non-ONFH) diagnosed at the Department of Orthopedics at our institution between January 1, 2011 and December 31, 2016 were retrospectively reviewed. The ONFH and non-ONFH groups comprised 488 and 496 cases, respectively. Basic information and peripheral blood cell levels of the two groups were compared.

**Results:**

The patients’ mean age was 59.89 ± 17.06 years (range: 38–82 years). There were 457 male and 527 female patients, with a male-to-female ratio of 1:1.15. We found that neutrophil granulocyte levels and percentage of neutrophil granulocytes were significantly different between the ONFH and non-ONFH groups. Multimodal regression analysis showed that the percentage of neutrophil granulocytes was an independent protective factor against ONFH.

**Conclusions:**

The factors influencing ONFH are neutrophil granulocyte levels and percentage of neutrophil granulocytes. Percentage of neutrophil granulocytes has a significant correlation with aseptic femoral head necrosis, providing a new perspective and direction for further study of femoral head necrosis.

## Background

Osteonecrosis of the femoral head (ONFH) is a disease with a high rate of disability. The cause of traumatic ONFH is clear, but that of non-traumatic ONFH is unknown. [1, 2] Main theories include the osteocyte apoptosis theory, abnormal lipid metabolism theory [3], and osteoporosis theory [4–6]. Even immunological factors play a role in ONFH development [7]. The common feature between these two conditions is that the blood supply in the femoral head is blocked by various causes, including mechanical and non-mechanical reasons, thereby resulting in osteonecrosis [8]. Studies have shown that intravascular micro thrombus formation of intravascular coagulation is the final stage of osteonecrosis [8, 9]. In the past, most studies focused on abnormal lipid metabolism and fat embolism, and there is less research on the correlation between peripheral blood neutrophil level and ONFH. Thus, this study aimed to explore the correlation between peripheral blood neutrophil levels and ONFH.

## Methods

### Subject selection

A total of 984 patients with ONFH and femoral neck fractures diagnosed at the Department of Orthopedics at our institution between January 1, 2011 and December 31, 2016 were retrospectively analyzed. Patients with complete laboratory examination results and those who had undergone peripheral blood examination early in the morning during admission were included in the analysis. Data on the erythrocyte sedimentation rate (ESR) (automatic biochemical analyzer, OLYMPUS 5421, Olympus Corporation, Tokyo, Japan and Hitachi-7600, Hitachi Corporation, Tokyo, Japan), C-reactive protein (CRP) (automatic biochemical analyzer, OLYMPUS 5421, Olympus Corporation, Tokyo, Japan), and V Classification Blood Routine Examination (five-class blood cell analyzer, SYSMEX XE-2100, SYSMEX CORPORATION Tokyo, Japan), and other indicators were collected. The exclusion criteria were as follows: patients with chronic medical diseases (coronary heart disease, chronic kidney disease, chronic obstructive pulmonary disease, cancer); history of alcohol abuse (average weekly consumption of pure alcohol content ≥500 ml per drinking episode); history of hormonal treatment, hip tuberculosis, congenital hip dysplasia, necrosis after hip infection, surgical treatment, and with missing basic information; and patients with both ONFH and femoral neck fractures treated before the first blood routine test. The included patients were divided into the following groups: the experimental group comprising patients with ONFH and the control group comprising patients with femoral neck fractures (non-ONFH).

### Data collection

Patients’ data, including age, sex, diagnosis, smoking history, drinking history (pure alcohol per week < 320 g), history of hormonal treatment, history of antihypertensive drug use, and history of hypoglycemic agent use, were obtained from the medical records. Detection data, including peripheral blood mononuclear macrophage level, neutrophil granulocyte level, percentage of neutrophil granulocytes, acid granulocyte level, percentage of acid granulocytes, alkaline granulocyte level, percentage of alkaline granulocytes, ESR, and CRP, were also collected.

### Statistical analysis

All statistical analyses were performed using SPSS 20.0 (IBM SPSS Statistics for Windows, Version 20.0. IBM Corp, Armonk, NY, USA). The measurement data were normally distributed and were presented as mean ± standard deviation. The independent samples t-test was used to compare the measurement data between the two groups. The count data were expressed as number of cases and proportions. The chi-squared test was used to compare count data between the two groups. Binary logistic regression was used to analyze the effect of these factors on ONFH. The test level α = 0.05 (*P* < 0.05) was considered significantly different.

## Results

A total of 984 patients were enrolled, with 488 patients in the ONFH group and 496 patients in the non-ONFH group. Patients’ mean age was 59.89 ± 17.06 years (range: 38–82 years). There were 457 male and 527 female patients, with a male-to-female ratio of 1:1.15.

Differences between mononuclear macrophage, neutrophil granulocyte, percentage of neutrophil granulocyte, acid granulocyte, percentage of acid granulocyte, alkaline granulocyte, percentage of alkaline granulocyte were statistically significant (Table 1). We grouped the neutrophil level and percentage of neutrophils in patients and found that the neutrophil level and percentage of neutrophils were correlated with the appearance of femoral head necrosis (Table 2). Multivariate regression analysis results showed that after adjusting for age, sex, and history of smoking, alcohol, hypertension, and diabetes, the percentage of neutrophil granulocytes was found to be an independent protective factor of ONFH (Table 3).

**Table 1:** Analysis of clinical basic characteristics of the two groups

Index	Non-Osteonecrosis N (%)	Osteonecrosis N (%)	X^2^/u	P
Mononuclear macrophage	0.50 (0.30,0.68)	0.36 (0.27,0.47)	−8.828	0.000
Percentage of mononuclear macrophages	6.30 (4.80,8.30)	6.10 (4.90,7.50)	−1.611	0.107
Neutrophil granulocyte	5.71 (4.14,7.25)	3.48 (2.78,4.49)	−15.357	0.000
Percentage of neutrophil granulocytes	76.20 (68.03,81.40)	60.20 (54.30,66.20)	−18.480	0.000
Acid granulocyte	0.08 (0.04,0.14)	0.14 (0.09,0.23)	−9.447	0.000
Percentage of acid granulocytes	1.30 (0.40,2.30)	2.40 (1.50,3.98)	−11.738	0.000
Alkaline granulocyte	0.02 (0.01,0.03)	0.02 (0.01,0.04)	−4.037	0.000
Percentage of alkaline granulocytes	0.30 (0.20,0.40)	0.40 (0.30,0.60)	−9.313	0.000
ESR	31.21 (28.78,33.90)	17.00 (9.00,25.00)	−15.197	0.000
C-reactive protein	32.78 (26.11,39.23)	4.64 (2.69,9.24)	− 22.211	0.000

**Table 2:** Single factor regression analysis of femoral head necrosis

Index	Statistics	Diagnosis
	N (%)	OR (95% CI) *P* value
Neutrophil granulocyte		
< 3.16	245 (24.90)	1
3.16-	246 (25.00)	0.576 (0.385,0.861) 0.007
4.36-	247 (25.10)	0.168 (0.113,0.250) < 0.0001
≥ 6.18	246 (25.00)	0.055 (0.035,0.086) < 0.0001
Percentage of neutrophil granulocyte		
< 59.00	244 (24.80)	1
59.00-	248 (25.20)	0.335 (0.213,0.527) < 0.0001
67.05-	246 (25.00)	0.073 (0.046,0.114) < 0.0001
≥ 77.88	246 (25.00)	0.022 (0.013,0.037) < 0.0001

**Table 3:** Multivariate Regression for Effect

Crude Model		Multivariate-Adjusted Model 1	Multivariate-Adjusted Model 2
OR (95% CI) *P* value		OR (95% CI) *P* value	OR (95% CI) *P* value
Neutrophil granulocyte			
< 3.16	1	1	1
3.16-	1.579 (0.919,2.713) 0.098	1.459 (0.838,2.538) 0.182	1.584 (0.893,2.809) 0.116
4.36-	1.320 (0.654,2.666) 0.438	1.191 (0.581,2.442) 0.633	1.373 (0.649,2.903) 0.407
≥ 6.18	1.485 (0.583,3.780) 0.407	1.294 (0.496,3.376) 0.599	1.611 (0.581,4.467) 0.359
Percentage of neutrophil granulocytes			
< 59.00	1	1	1
59.00-	0.316 (0.189,0.527) < 0.0001	0.287 (0.169,0.488) < 0.0001	0.275 (0.159,0.476) < 0.0001
67.05-	0.062 (0.034,0.113) < 0.0001	0.056 (0.030,0.104) < 0.0001	0.054 (0.028,0.103) < 0.0001
≥ 77.88	0.021 (0.009,0.047) < 0.0001	0.018 (0.008,0.042) < 0.0001	0.019 (0.008,0.045) < 0.0001

Crude Model adjusted for: none;

Multivariate-Adjusted Model 1 adjusted for: age; sex; smoking; alcohol use; hypertension; diabetes;

Multivariate-Adjusted Model 2 adjusted for: age; sex; smoking; alcohol use; hypertension; diabetes; hormone; hypertension

## Discussion

In this study, we investigated the correlation between neutrophil granulocyte levels in the peripheral blood and ONFH. Our study results reveal that the mononuclear macrophage level, percentage of mononuclear macrophages, neutrophil granulocyte level, percentage of neutrophil granulocytes, acid granulocyte level, percentage of acid granulocytes, and percentage of alkaline granulocytes were associated with ONFH. Moreover, mononuclear macrophage levels and neutrophil percentages were associated with a significantly higher incidence of femoral head necrosis.

ONFH is caused by a variety of factors that lead to avascular necrosis, deformation, collapse of the femoral head, and loss of hip function [10]. The femoral head necrosis rate is extremely high, which increases the burden on society and patients. A study of 30,030 patients aged 15 years and older from 9 provinces in China found that 218 patients were diagnosed as having non-traumatic ONFH, which is equivalent to approximately 9.92 million people [11]. In China, the cost of initial total hip arthroplasty ranges from $1000 to $8000, which imposes an enormous economic and social burden on patients and the state. There are many factors that contribute to femoral head necrosis. The main theories include blood stagnation [12, 13], lipid metabolism abnormality [3], bone cell apoptosis [14], and osteoporosis, to name a few. The specific etiological mechanism is unknown, which makes early diagnosis and treatment of ONFH difficult.

In this paper, we found that age, sex, hypertension, diabetes, and other factors were statistically significantly correlated to ONFH, for several possible reasons. First, in this study, patients with femoral neck fractures were considered to have no bone necrosis, i.e., the control group. Given that femoral neck fractures occur mostly in the elderly and women, which is commonly due to postmenopausal osteoporosis, the difference is greater [15]. Second, elderly patients often have underlying diseases, such as hypertension and diabetes, whereas the ONFH group is relatively young and had fewer patients with hypertension and diabetes; thus, differences were noted between the groups.

Neutrophils are cells that are produced in the bone marrow and play important immune functions in the systemic circulation. They are important barriers for the body’s defense against invasive factors and constitute an important component of the body’s natural immunity [16]. Many studies have shown that inflammation is associated with the prognosis of a variety of diseases throughout the body [17]. Gao et al. found that the ratio of neutrophils to lymphocytes is related to the prognosis of pancreatic cancer [18]. Lattanzi et al. reported that neutrophils-lymphocyte ratio (NLR) is associated with the prognosis of acute cerebral hemorrhage [19]. Moreover, Tan et al. found that NLR is an independent predictor of cardiovascular morbidity [20]. Swierczak et al. suggested that neutrophils have a certain correlation with malignant tumor metastasis [21]. The data from this study suggest that neutrophil levels are associated with the development of ONFH. Moreover, neutrophil levels may play an important role in the pathogenesis of ONFH.

Many researchers have recognized that there are several imbalances in ONFH, such as osteoblast apoptosis, and an imbalance in bone formation and bone resorption, resulting in osteoporosis of the subchondral bone of the femoral head, which eventually leads to necrosis of the femoral head [8, 22]. Some scholars have found through animal experiments that glucocorticoids cause apoptosis of bone cells and osteoblasts by reducing the expression of β-catenin and c-Myc downstream of the Wnt pathway, leading to early steroid-induced femoral head necrosis [23]. The expression of vascular endothelial growth factor and BMP2 in bone tissue decreased, and the BMP2/Drosophila MAD protein/Runx2 signaling pathway was inhibited, thereby inhibiting the differentiation of bone marrow stromal cells into osteoblasts, promoting their differentiation into osteoclasts, and affecting the structure of the femoral head. Destruction and osteoporosis eventually lead to ONFH [24]. In addition, inflammation is closely related to osteoporosis, in which IL-1β, TNF-α, and IL-6 can activate the macrophages of the NF-κB pathway, thereby causing them to differentiate into osteoclasts. As a result, bone resorption increases, leading to early osteoporosis and micro fracture in the femoral head, which in turn leads to osteonecrosis [25, 26].

Femoral head necrosis is essentially a sterile inflammatory change. Studies have suggested that inflammatory bone loss in inflammatory diseases is associated with increased activation of osteoclasts by receptor activation of NF-KB ligand (RANKL) and that neutrophil granulocytes, which are present during osteolysis and in adjacent sites of inflammation, are the main infiltrating cells in this process [27]. Poubelle et al. believes that neutrophils have the ability to express RANKL and OPG and participate in local immune responses and bone remodeling processes [28]. Additionally, through the molecular pathway research of *Salvia miltiorrhiza*, Molecular pathway research of Salvia miltiorrhiza reveals that it mainly affects Wnt, NF-κB, HIF-1, MAPK, Notch, PI3K-Akt, and other signaling pathways closely related to ONFH. Pathways can be divided into the following broad categories: promoting bone formation of osteoblasts and inhibiting bone resorption by osteoclasts. This provides evidence that femoral head necrosis is associated with bone formation and absorption [29–31]. Neutrophil levels may be associated with inhibition of osteoclast formation and reduction of bone resorption, leading to femoral head necrosis. This supports the correlation between neutrophil levels and femoral head necrosis revealed in our study data.

A large number of related pathological studies have confirmed that osteonecrosis and bone remodeling coexist in the early stage of femoral head necrosis [32–34]. It is indicated that the bone reconstruction process is of great significance in reversing the early necrosis of the femoral head or delaying the process of osteonecrosis. These data suggest that the speed of femoral head necrosis is related to the active immune defense and necrotic tissue cleansing of neutrophils in the early stage of osteonecrosis. More importantly, neutrophils infiltrate in the early stage of femoral head necrosis and participate in the bone remodeling process. In addition, neutrophils release high levels of MMP9 under the stimulation of certain factors to regulate the activity of other proteases and cytokines and promote the release of vascular endothelial growth factor (VEGF). VEGF is a homodimeric glycoprotein that was first purified from the follicular stellate cells of the bovine pituitary by Ferarara in 1989 [35], and it is the most effective angiogenic factor. It can exert a strong role in promoting endothelial proliferation angiogenesis and revascularization; revascularization is important for the outcome of early femoral head necrosis [36].

This study has several limitations. First, the sample size is not large enough, as the multi-center research data are lacking. Second, the specific etiology and pathogenesis of ONFH are still in the exploration stage. Third, the personal factors of the doctors and patients may have an influence on the data results, which may have a clinical impact on the research.

## Conclusion

Percentage of neutrophil granulocytes has a significant correlation with aseptic femoral head necrosis, providing a new perspective and direction for further study of femoral head necrosis.

## Data Availability

Data from the Department of Orthopedics, the First Affiliated Hospital of Nanchang University, Artificial Joints Engineering and Technology Research Center of Jiangxi Province, Nanchang, Jiangxi, China. The datasets used and/or analyzed during the current study are available from the corresponding author on reasonable request.

## Abbreviations

BMP: Bone morphogenetic Protein
CRP: C-reactive protein
ESR: Erythrocyte sedimentation rate
NLR: Neutrophil-lymphocyte ratio
ONFH: Osteonecrosis of femoral head
VEGF: Vascular endothelial growth factor
